# Case Report: Pneumothorax and Pneumomediastinum as Uncommon Complications of COVID-19 Pneumonia—Literature Review

**DOI:** 10.4269/ajtmh.20-0815

**Published:** 2020-07-23

**Authors:** Alvaro Quincho-Lopez, Dania L. Quincho-Lopez, Fernando D. Hurtado-Medina

**Affiliations:** 1 San Fernando Medical School, Universidad Nacional Mayor de San Marcos, Lima, Peru;; 2 Diagnostic Imaging Service, Hospital II LNC Luis Negreiros Vega ESSALUD, Lima, Peru

## Abstract

As the COVID-19 pandemic progresses, awareness of uncommon presentations of the disease increases. Such is the case with pneumothorax and pneumomediastinum. Recent evidence suggested that these can occur in the context of COVID-19 pneumonia, even in the absence of mechanical ventilation–related barotrauma. We present two patients with COVID-19 pneumonia complicated by pneumomediastinum. The first patient was a 55-year-old woman who developed COVID-19 pneumonia. Her clinical course was complicated by pneumothorax and pneumomediastinum, and, unfortunately, she died 2 days following the admission. The second patient was a 31-year-old man who developed a small pneumomediastinum and was managed conservatively. He had a spontaneous resolution of the pneumomediastinum and was discharged 19 days later. None of our patients required invasive or noninvasive positive pressure ventilation. We performed a literature review of COVID-19 pneumonia cases that developed pneumothorax, pneumomediastinum, or both. The analysis showed that the latter had high mortality (60%). Thus, it is necessary to pay attention to these complications as early identification and management can reduce the associated morbidity and mortality.

## INTRODUCTION

Both pneumothorax and pneumomediastinum are known complications of mechanical ventilation due to intubation.^1,2^ Nonetheless, even without barotrauma involved, pneumothorax or pneumomediastinum, or more rarely both, can be present in the context of COVID-19.^3,4^ Herein, we report two cases of patients infected with SARS-CoV-2, who developed pneumomediastinum, and one of them also presented pneumothorax. We also performed a relevant literature review using the Scopus database.

## CASE DESCRIPTIONS

**Figure 1.**
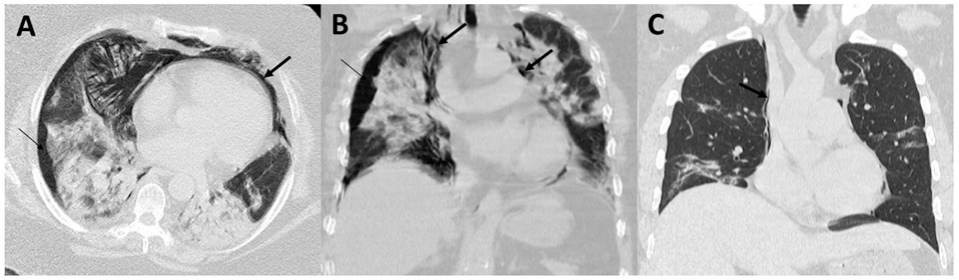
(**A**) Axial image and (**B**) coronal reconstruction showing pneumothorax (thin arrow) and pneumomediastinum (thick arrow) in case 1. (**C**) Axial image showing a small pneumomediastinum (thick arrow) in case 2.

The first case was a 55-year-old woman with a past medical history of hypertension, uncontrolled bronchial asthma interspersed with periods of inactivity, and morbid obesity. She presented to the emergency department (ED) with 7 days of marked dyspnea, chest pain, and dry cough. Previously, she received outpatient treatment with prednisone and dexamethasone every 8 hours for 5 days. On admission, her vital signs showed tachypnea (22 breaths/minute), with high temperature (38.2°C), increased heart rate (110 beats/minute), and 85% saturation. On physical examination, she had bilateral basal crackles and peripheral cyanosis. Laboratory results showed an elevated C-reactive protein (CRP) of 2.66 mg/dL (normal range 0–0.50 mg/dL). Her blood count showed leukocytosis (30,270 cells/μL) with a lymphocyte count of 1,210 cells/μL. The patient was reactive to the COVID-19 IgG/IgM rapid test. Non-contrast chest computed tomography (CT) showed some ground-glass opacities of peripheral subpleural location, associated with multiple areas of consolidation in posterior segments of both lower lobes, with the presence of pneumothorax (approximately 20%) and pneumomediastinum (Figure 1A and B). She received treatment with azithromycin, ceftriaxone, hydrocortisone, and supplemental oxygen with a reservoir mask. She did not receive noninvasive positive pressure ventilation. The pneumothorax and pneumomediastinum were managed conservatively. However, despite the support measures, the patient died from respiratory failure 2 days after admission.

The second case was a 31-year-old man with a past medical history of chronic gastritis and hypercholesterolemia in control, who presented to the ED with 6 days of dyspnea, general malaise, dry cough, and continuous fever for 4 days. On admission, tachypnea (22 breaths/minute), high temperature (38.5°C), normal heart rate (94 beats/minute), and saturation of 94% were noted. Physical examination revealed bibasal crackles. He did not receive noninvasive positive pressure ventilation. Laboratory results showed an elevation of CRP of 1.84 mg/dL (normal range 0–0.50 mg/dL). His blood count showed elevated white blood cells (18,270 cells/μL [normal range 3,980–10,040 cells/μL]) with a lymphocyte count of 1,077 cells/μL. The patient was reactive to the IgG/IgM rapid test, receiving treatment with azithromycin, ceftriaxone, hydrocortisone, and supplemental oxygen with nasal cannula. A new CT was performed because of desaturation after the removal of oxygen support from the patient (on day 15 of hospitalization) and showed some foci of consolidation in posterior segments of both lower lobes, associated with parenchymal bands in both hemithoraces, with the presence of laminar air content predominantly on the right side, consistent with pneumomediastinum (Figure 1C). The patient remained hospitalized for 19 days. A conservative management was chosen because the pneumomediastinum was very small, and its resolution was observed in the subsequent control after 7 days of discharge.

## DISCUSSION

The symptoms of SARS-CoV-2 infection have been widely characterized in large studies, with fever, cough, and dyspnea being the most frequent. These same studies indicate that only 1–2% of patients developed pneumothorax^5,6^; although it may occur as the disease progresses,^3^ its presentation is still infrequent, like pneumomediastinum.^4^ The mechanism is not fully elucidated, although it is probably because of rupture of the alveolar wall due to the increasing pressure difference between the alveolus and the pulmonary interstitium.^7,8^

Pneumothorax and pneumomediastinum are defined as the presence of free air in the pleural and mediastinal cavities, respectively.^9,10^ Spontaneous pneumothorax can be primary or secondary, depending on the absence or presence of an underlying lung disease.^11^ By contrast, pneumomediastinum can be primary, or spontaneous, if the cause is idiopathic, or secondary if it responds to a known etiology, whether traumatic or iatrogenic.^10^ Chest pain and dyspnea are the most common symptoms.^11,12^ An important difference is that pneumothorax occurs mainly at rest,^9^ whereas strenuous physical activity has been reported as a triggering event for developing pneumomediastinum.^12^ However, both are more frequent in males.^9,12^ Drug abuse, asthma, and other lung diseases such as chronic obstructive pulmonary disease and interstitial lung disease are some predisposing factors, with tobacco being the most important risk factor in both.^9,10^ None of our patients were active smokers, and only the first one had asthma as a risk factor in developing one of the two complications.

Chest CT as a diagnostic test to identify patients with COVID-19 has a high sensitivity and a high negative predictive value.^13^ Moreover, depending on the lung involvement, it shows different phases: the early phase, where the ground-glass pattern of subpleural distribution uni- or bilateral predominates; the progression phase, where, in addition to ground-glass involvement, there are also paved areas or “crazy paving” with diffuse or multi-lobar distribution; the peak, or the most affected phase, where the affected areas progressively consolidate; and, finally, the absorption phase, where ground glass appears secondary to the absorption of consolidations.^14^

Table 1 presents a summary of case reports of patients infected with SARS-CoV-2 who presented pneumothorax, pneumomediastinum, or both. Seventeen reports describing 20 patients were found. In total, half of the patients presented a favorable evolution (50%; 10/20), whereas 30% (6/20) died. Follow-up was not reported in four patients. It is important to note that subcutaneous emphysema was a radiological finding present in 35% (7/20) of the patients, two in pneumothorax,^15,16^ three in pneumomediastinum,^4,7,17^ and two in both.^18,19^ The treatment for COVID-19 was variable. Nevertheless, for the management of pneumothorax or pneumomediastinum, some patients (40%; 8/20) required a chest tube drainage,^8,16,19–24^ others (5%; 1/20) also required needle aspiration,^24^ and thoracoscopy and bleb resection were required in two (10%) cases of persistent or recurrent pneumothorax.^25^ The remaining patients were either managed conservatively or not reported.

**Table 1:** Summary of pneumothorax or/and pneumomediastinum in patients infected with SARS-CoV-2

Reference	Country	Gender/age (years)	Comorbidities	Symptoms	Radiological findings	Complications	Onset of symptoms (days) until outcome	Management	Outcome
Pneumothorax									
Aiolfi et al.^25^	Italy	M/56	Active smoking	Fever, cough, and respiratory distress	CT: bilateral, peripheral GGOs. CXR: left-side pneumothorax	None	12+	Intubation at the ICU. Three-port left-side thoracoscopy and bleb resection	Favorable
Aiolfi et al.^25^	Italy	M/70	None	Fever, fatigue, and respiratory distress	CT: bilateral, subpleural GGOs. CXR: left-side pneumothorax	None	5+	Intubation at the ICU. Three-port left-side thoracoscopy and bleb resection	Favorable
Corrêa Neto et al.^20^	Brazil	F/80	Hypertension and ischemic heart disease, currently using clopidogrel, aspirin, losartan, and carvedilol	Dry and persistent cough, fever, SOB, and diffuse abdominal pain	CT: ground-glass pattern in the bilateral pulmonary parenchyma, pneumothorax on the right, and extensive pneumoperitoneum, with free intracavitary fluid	Refractory septic shock	12+	Hydration, orotracheal intubation, broad-spectrum antibiotic therapy, and right chest drainage	Died
Flower et al.^24^	United Kingdom	M/36	Childhood asthma and a 10-pack-year history of smoking	Fever, dry cough, SOB, and left-sided pleuritic chest pain	CXR: large left-sided pneumothorax with mediastinal shift and radiological signs of tension. The right lung displayed widespread patchy consolidative changes. CT: widespread areas of patchy consolidation, with associated bullae	None	23	Emergency needle decompression, and a chest drain	Favorable, discharged after 2 days
Hollingshead et al.^21^	USA	M/50	NR	SOB	CT: diffuse GGOs throughout the chest but also a 10-cm loculated posterior right pneumothorax	None	NR	Chest tube	Favorable
Rohailla et al.^22^	Canada	M/26	None	Sudden-onset right-sided pleuritic chest pain and progressive SOB	CXR: large right pneumothorax with complete collapse of the right lung without mediastinal shift	None	5	Small catheter chest drain	Favorable, discharged after 2 days
Spiro et al.^23^	Germany	M/47	Splenectomy, and HIV under antiretroviral therapy	Fever, dry cough, SOB, and stenocardia	CT bipulmonary GGOs and consolidations with a multi-lobar, peripheral, and dorsal distribution. Right-sided tension pneumothorax	None	34	Morphine and azithromycin. Then, chest tube through open thoracotomy	Favorable, discharged after 20 days
Sun et al.^15^	China	M/38	None	Fever and cough	CT: GGOs in the left lower lobe. Then, lesions turned into consolidation. Emphysema, giant bulla, small pneumothorax, and pleural effusion	Dyspnea and severe hypoxemia. Acute respiratory distress syndrome	34+	High-flow nasal cannula. Then, noninvasive mechanical ventilation at the ICU	NR
Xiang et al.^16^	China	M/67	Coronary artery bypass, chronic pulmonary diseases (obsolete pulmonary tuberculosis, chronic bronchitis, and emphysema)	Dyspnea, fatigue, and mild diarrhea	CXR: extensive air-space opacities in bilateral lungs. Subcutaneous emphysema, mediastinal emphysema and a small amount of pneumothorax on both sides	Sinus bradycardia and left ventricular enlargement with ejection fraction 20%	27	Invasive ventilation at the ICU. Then, prone position ventilation, vasoconstrictor, antibacterial, and antiviral therapy. Chest closed drainage	Died, 12 days after admission
Pneumomediastinum									
Kolani et al.^27^	Morocco	F/23	NR	asymptomatic	CT: inconspicuous GGOs in the lower left inferior lobe and a small amount of air in the mediastinum without any fluid infiltration	None	10+	Azithromycin and chloroquine	Favorable, discharged after 10 days
Lacroix et al.^7^	France	M/57	None	severe acute dyspnea, fever, cough, breathlessness, diarrhea, and anosmia	CXR: diffuse subcutaneous emphysema and bilateral consolidations. CT: pneumomediastinum, subcutaneous emphysema, consolidations, GGOs and crazy paving	NR	14+	Intubation for mechanical ventilation	NR
Lei et al.^28^	China	M/64	NR	Fever and fatigue	CT: progressive resolution of the patient’s pneumonic lesions and spontaneous pneumomediastinum in the anterior mediastinum	None	24+	NR	NR
Mohan et al.^17^	USA	M/49	Hypertension and type 2 diabetes	Fever, cough, SOB, and anosmia	CXR: bilateral patchy infiltrates. CT: severe pneumomediastinum with extensive subcutaneous emphysema	Nausea and vomiting	18	Ceftriaxone, doxycycline, steroids, enoxaparin sodium, and hydroxychloroquine and noninvasive supplemental oxygen	Favorable, discharged after 15 days
Wang et al.^29^	China	F/36	Mastitis	Fever, cough, and bloody sputum, and SOB	CT: multiple diffuse patchy consolidation areas and GGOs in both lungs. There was interlobular septal thickening with pleural effusion and bronchiectasis	Respiratory failure and acute respiratory distress syndrome	14	Combined antiviral drugs, anti-inflammatory drugs, and supportive care	Died after 2 days
Zhou et al.^4^	China	M/38	NR	Fever, cough, and headaches	CT: multiple GGOs with bilateral parenchymal consolidation and interlobular septal thickening, subcutaneous emphysema, and pneumomediastinum	SOB and exertional angina	31	Broad-spectrum antibiotic therapy, recombinant human interferon alfa-1b, and supplemental oxygen	Favorable, discharged after 30 days
Pneumothorax and pneumomediastinum									
López Vega et al.^8^	Spain	F/84	Anticoagulation due to prosthetic valve replacement, renal failure, stage C heart failure with preserved ejection fraction, hypertension, and hypercholesterolemia	Fever, cough, and dyspnea	CT: right partial hydropneumothorax, left full hydropneumothorax, and pneumomediastinum. Pulmonary involvement by COVID-19	Progressive deterioration of respiratory function, atelectasis of the left lung until developing a white lung	18	Hydroxychloroquine, ceftriaxone, methylprednisolone, and oxygen supplementation	Died
López Vega et al.^8^	Spain	M/67	NR	Fever and dyspnea	CXR: bilateral opacities with multi-lobar affectation. CT: a pneumothorax chamber and pneumomediastinum	Decreased renal function, worsening respiratory function, and multiple organ failure	18	Piperacillin/tazobactam and azithromycin at the ICU, and pleural drainage tube	Died
López Vega et al.^8^	Spain	M/73	Basal cell epithelioma, obstructive sleep apnea, obesity, and depression under pharmacological treatment	Fever and dyspnea	CXR: alveolar opacity with a bibasal air bronchogram. CT: extensive bilateral involvement by coronavirus, and a minimal chamber of pneumomediastinum	Progressive deterioration	20	Hydroxychloroquine, azithromycin, tocilizumab, methylprednisolone, and oxygen support. Then, anticoagulant therapy and noninvasive ventilatory support with continuous positive airway pressure	Died 15 days after admission
Ucpinar et al.^19^	Turkey	F/82	None	Fever, SOB, and persistent cough	CT: widespread bilateral GGOs, predominantly in lower lobes. In addition, pneumomediastinum, left-sided massive pneumothorax, and subcutaneous emphysema in the neck posterior thoracic wall were identified	None	11+	Hydroxychloroquine, oseltamivir, ceftriaxone, and a chest tube	Favorable, discharged after 11 days
Wang et al.^18^	China	M/62	None	Fever, cough, and dyspnea	CT: multiple GGOs with parenchymal consolidation, pneumothorax on the right, combined with pneumomediastinum, and subcutaneous emphysema	None	47	Oxygen therapy, lopinavir/ritonavir, as well as antibiotics and steroid therapy	Favorable, discharged

CT = computed tomography; CXR = chest X-ray; GGOs = ground-glass opacities; ICU = intensive care unit; NR = no reported; SOB = shortness of breath.

In those patients with pneumothorax, the majority were male (88.8%; 8/9), and 55.5% (5/9) had some comorbidities. Fever was the most frequent symptom on admission (66.6%; 6/9). Furthermore, the majority presented a favorable evolution (55.5%; 5/9), with those with the highest number of associated comorbidities having the worst evolution (22.2%; 2/9). Follow-up was not reported in one patient. Pneumothorax may also present as a late sequel to COVID-19.^21^ Although most cases report spontaneous pneumothorax, tension pneumothorax is also a possible complication.^23,24^ Bulla associated with pneumothorax is reported in two patients.^15,24^ Some authors consider the rupture of a bulla to be the cause of spontaneous pneumothorax.^9^

Of the pneumomediastinum cases, male gender was the most affected (66.6%; 4/6), and only 33.3% (2/6) presented any associated comorbidity. However, in 50% of the cases, the risk factors were not reported. In 83.3% (5/6), fever was reported as the most frequent symptom, and one patient did not present any symptoms. The evolution was favorable in 50% of the cases.

Of the patients who presented pneumothorax and pneumomediastinum at the same time, most of the patients were male (60%; 3/5), and 40% (2/5) had some associated comorbidity. Fever was the most frequent symptom (100%), followed by dyspnea (80%) and cough (60%). Death was inevitable in 60% (3/5) of the patients.

Some limitations that we can point out are that certain articles that are not indexed in Scopus could not be included in our review. However, we decided to use Scopus because it contains all the documents included in MEDLINE,^26^ ensuring not only quantity but also quality of the documents. Furthermore, we only considered articles written in English. Our literature review was last updated on July 3, finding 32 results, of which only 17 are case reports. To enable the reproducibility of our review, we display our search query: (TITLE-ABS {“2019-nCoV” OR “COVID-19” OR “NCOVID-19” OR “HCoV-19” OR “SARS-nCoV” OR “SARS-CoV-2” OR “severe acute respiratory syndrome coronavirus 2”} OR TITLE-ABS {coronavirus W/2 [wuhan OR china OR novel OR pneumonia]}) AND (TITLE-ABS [pneumomediastinum OR pneumothorax]) AND (LIMIT-TO [PUBYEAR, 2020]).

In conclusion, pneumothorax and pneumomediastinum are possible complications of COVID-19 pneumonia, causing acute decompensation that can worsen the prognosis of patients, especially those with underlying lung diseases.
